# The calcium channel β2 (CACNB2) subunit repertoire in teleosts

**DOI:** 10.1186/1471-2199-9-38

**Published:** 2008-04-17

**Authors:** Alicia M Ebert, Catherine A McAnelly, Ashok Srinivasan, Rachel Lockridge Mueller, David B Garrity, Deborah M Garrity

**Affiliations:** 1 Department of Biology, Colorado State University, Fort Collins, CO 80523, USA; 2 NIH/NHLBI, 10 Center Drive, Rm. 6C/104, Bethesda, MD 20892, USA

## Abstract

**Background:**

Cardiomyocyte contraction is initiated by influx of extracellular calcium through voltage-gated calcium channels. These oligomeric channels utilize auxiliary β subunits to chaperone the pore-forming α subunit to the plasma membrane, and to modulate channel electrophysiology [1]. Several β subunit family members are detected by RT-PCR in the embryonic heart. Null mutations in mouse β2, but not in the other three β family members, are embryonic lethal at E10.5 due to defects in cardiac contractility [2]. However, a drawback of the mouse model is that embryonic heart rhythm is difficult to study in live embryos due to their intra-uterine development. Moreover, phenotypes may be obscured by secondary effects of hypoxia. As a first step towards developing a model for contributions of β subunits to the onset of embryonic heart rhythm, we characterized the structure and expression of β2 subunits in zebrafish and other teleosts.

**Results:**

Cloning of two zebrafish β2 subunit genes (β2.1 and β2.2) indicated they are membrane-associated guanylate kinase (MAGUK)-family genes. Zebrafish β2 genes show high conservation with mammals within the SH3 and guanylate kinase domains that comprise the "core" of MAGUK proteins, but β2.2 is much more divergent in sequence than β2.1. Alternative splicing occurs at the N-terminus and within the internal HOOK domain. In both β2 genes, alternative short ATG-containing first exons are separated by some of the largest introns in the genome, suggesting that individual transcript variants could be subject to independent cis-regulatory control. In the *Tetraodon nigrovidis *and *Fugu rubripes *genomes, we identified single β2 subunit gene loci. Comparative analysis of the teleost and human β2 loci indicates that the short 5' exon sequences are highly conserved. A subset of 5' exons appear to be unique to teleost genomes, while others are shared with mammals. Alternative splicing is temporally and spatially regulated in embryo and adult. Moreover, a different subset of spliced β2 transcript variants is detected in the embryonic heart compared to the adult.

**Conclusion:**

These studies refine our understanding of β2 subunit diversity arising from alternative splicing, and provide the groundwork for functional analysis of β2 subunit diversity in the embryonic heart.

## Background

Voltage-dependent L-type Ca^2+ ^channels (V-LTCC) are essential for the initiation and regulation of excitation-contraction coupling in cardiac muscle. In addition, Ca^2+ ^entry through V-LTCC channels can also serve as a second messenger to modulate regulatory protein kinases, calmodulin and beta-adrenergic responses [3]. Genetic mutation of key Ca^2+ ^homeostasis proteins is frequently associated with defects in both cardiac cell differentiation and contractility, suggesting Ca^2+ ^signaling is essential for normal cardiac development [4-7]. Ca^2+ ^signals can also contribute to the control of gene expression [8]. Ca^2+ ^influx through V-LTCC can dramatically affect the phosphorylation, activity, or expression of many genes [5,8-10]. These data support the hypothesis that Ca^2+ ^signaling through V-LTCC impacts not only cardiac contraction but also embryonic cardiac growth and morphology, physiology, and gene expression.

V-LTCCs are oligomeric proteins composed of a pore-forming α1 subunit and up to four auxiliary subunits, termed α2, β, γ, and δ, associated in a 1:1:1:1 stoichiometry [11,12]. Mammals encode 10 distinct α1 subunit genes (four of which form V-LTCC) and four distinct β subunit genes (β1 – β4). Particular α1 and β subtypes are able to associate non-exclusively to form heterogeneous channels [13-17]. V-LTCC that contain the α1c subunit (Ca_v_1.2) constitute the vast majority of L-type channels expressed in adult cardiac muscle [17-22].

The V-LTCC β subunit genes have important functions in cardiac, skeletal and smooth muscle, the central nervous system and the retina [2,23-28]. β subunits modulate Ca^2+ ^channel function in two distinct ways. First, they facilitate transport of the α1 to the plasmalemma. In addition, they interact with α1 to modulate both the kinetics and voltage-sensitivity of the channels [29-31]. As members of the membrane-associated guanylate kinase (MAGUK) family, the V-LTCC β subunit genes include conserved SH3 and guanylate kinase (GK) domains connected by a bridging region termed the HOOK domain [32]. The larger MAGUK protein family appears to be specific to metazoans, and its core structure, including the SH3 and GK domains, can be traced back to early metazoan history [33]. All known β genes undergo alternative splicing of 5' exons, and some exhibit alternative splicing of internal exons as well [34-36]. Alternative splicing in internal exons can create truncated proteins, some of which have been shown to mediate calcium channel-independent functions in the cell [1,36,37].

In the mouse, the earliest documented expression of a cardiac β protein is a Western blot showing β2 protein in the linear heart tube (E8.5) [2]. By E9.5 (chamber morphogenesis stages), β4 and β2 mRNA and protein are clearly present in the embryonic myocardium [2,10,38]. β2 and β4 proteins show detectable differences in spatial distribution as early as E9.5, which become more pronounced in mid- to late embryogenesis (E10.5 – E15.5) and in later fetal development [38]. In rat postnatal development (4.5 weeks), RT-PCR studies indicate all four β subunits are expressed in cardiac tissue, though they differ in abundance [39]. Significantly, by 4.5 weeks RNA from atria and ventricles show different β subunit RT-PCR expression profiles [39]. In brain and heart, newborn and juvenile animals express different subsets of β genes (including different transcript variants) than the adult [39,40]. The physiological significance of these observations is not known. However, a recent report describing the targeted mutagenesis of mouse β2 indicated that homozygous loss of this gene (β2^-/-^) is embryonic lethal at E10.5, significantly earlier than the α1C-deficient mouse [2,19]. No up-regulation of other β subunits was observed in β2^-/- ^mutants. In β2^-/- ^mutant mice, cardiac contractility is severely compromised and V-LTCC currents diminished. Hearts in mutant mice exhibit severe bradycardia and vascular patterning defects, but these phenotypes may be secondary to severe morphological defects that develop in the heart or to the overall reduced embryonic growth [2]. In contrast to β2, mice with null mutations in β1, β3 and β4 have no reported cardiac phenotypes [23,25-28]. However, the zebrafish β1^-/- ^mutant *relaxed *develops pericardial edema and reduced circulation at 4 dpf (days post-fertilization), suggesting a possible compromise in cardiac function [41]. Thus, while multiple β subunits are clearly present in the embryonic heart, much remains to be learned about their functional roles in development.

As a model for the study of β subunit developmental function in the heart, zebrafish has several advantages, including the external fertilization of embryos and rapid, transparent development of the larvae, which allows experimental access to the developing heart and circulatory system. Moreover, for about the first 6 days of development the small zebrafish larvae subsist on oxygen acquired via diffusion, enabling them to survive without a functional heart [42]. As a first step in establishing a zebrafish model, we cloned two β2 subunit gene homologs from zebrafish. We find one β2 subunit is much more similar than the other to known vertebrate β2 subunits. Significant alternative splicing occurs involving several ATG-containing first exons in both zebrafish genes, as well as two other teleost species. The separation of these short exons by extremely large introns suggests potential independent cis-regulatory control of alternatively spliced transcripts. Unexpectedly, both β2 subunits were maternally expressed, consistent with possible early roles for this gene in morphogenetic movements or patterning. Additionally, both genes showed evidence of tissue-, stage- and transcript-specific regulatory control in embryos and adults.

## Results

### Identification of zebrafish and pufferfish calcium channel β2 subunit (CACNB2) genes

**Figure 1:**
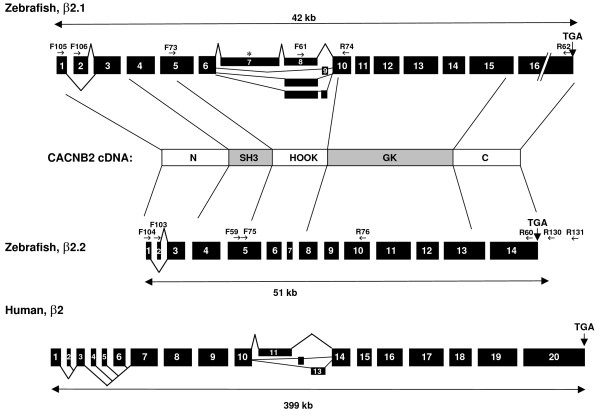
**Structure of zebrafish β2.1 and β2.2 MAGUK genes**. Exons are shown as black boxes; introns are not drawn to scale. Alternative splicing is indicated in the N-terminus and HOOK domain-encoding regions. * indicates the site of a premature stop codon in β2.1 transcripts that include exon 7. Labeled arrows indicate the locations and names of primers used in RACE and RT-PCR. Human exon structure was adapted from [57]. See Additional File 2A for primer sequences.

**Figure 2:**
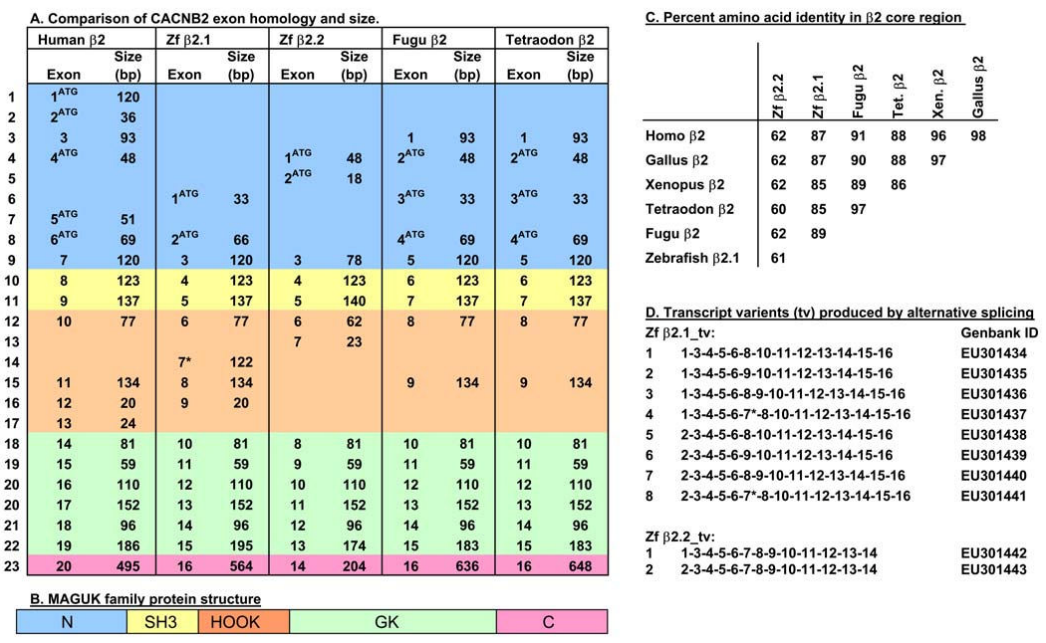
**Comparative gene structure in teleost β2 subunits**. A) The exons identified for each of four teleost β2 genes are listed vertically with indication of their size in bp. Exons on each horizontal line of the table are homologous in sequence (see later figures for alignments). Exons are color coded to indicate what portion of the 5-domain MAGUK protein (diagrammed in B) they comprise. ^ATG^, indicates the presence of an initiation codon in the exon. *, indicates the exon contains a premature in-frame termination codon. C) Percent amino acid identity among vertebrate β2 subunits within the SH3 through GK sequences. D) The transcript variant numbers assigned to the alternatively spliced transcripts are listed on the left; transcript variant composition is depicted by exon numbers.

TBLASTN searches of the GenBank database at NCBI using human β2 subunit sequences suggested that zebrafish encode two β2 homologues [43]. We therefore designed primers to highly conserved sequences within the SH3 and GK protein domains, and performed 5' and 3' RACE-PCR on RNA extracted from embryos aged 1–3 dpf. Using RACE and reverse-transcriptase PCR (RT-PCR), we isolated cDNAs representing two zebrafish β2 genes, termed β2.1 and β2.2 (see Figs. 1, 2 and Additional File 1). The β2.1 gene showed a near perfect match with genomic sequences located within Genbank zebrafish chromosome 22 (NC_007133.1), whereas β2.2 matched genomic sequences found on chromosome 2 (NC_007113.1) [43-45]. Alternative splicing occurred at the N-terminus for both genes and within the HOOK domain for β2.1 (see Figs. 1 and 2D for descriptions of transcript variants). In the initial RACE analysis for β2.1, we recovered four β2.1_tv1 clones, and five β2.1_tv6 clones, suggesting these may be the most abundant transcript variants. One clone each was found for the β2.1_tv2, 3, 4, 5, 7 and 8 transcripts, suggesting they may be less abundant. For β2.2, we recovered two β2.2_tv1 and three β2.2_tv2 5' RACE clones. In additional RT-PCR analysis using primers closely flanking the HOOK domain, we confirmed that β2.1 transcript variants containing more than one exon between exons 6 and 10 (i.e. β2.1_tv3, 4, 7 and 8) could be detected in the RNA samples (see Additional file 2). Conversely, we confirmed that no alternative splicing occurred in the β2.2 HOOK domain (see Additional File 2). Several 3' RACE clones were of a single variant for β2.1 and a single variant for β2.2, suggesting that no alternative splicing occurred in the GK or C-terminal regions of the genes.

To better assess the conservation of the new genes, we performed a comparative search for orthologous β2 sequences in the pufferfish *Tetraodon nigrovidis *and *Fugu rubripes *genomes [45-47]. We identified a single β2 subunit gene in each pufferfish genome (Fig. 2A). All four teleost β2 genes contained highly conserved SH3 and GK domains characteristic of MAGUK family proteins (Fig. 2B). The size and number of exons comprising these domains are nearly identical in teleosts and mammals (Fig. 2A, yellow and green sections). Pairwise comparisons of amino acid sequences within these core (SH3 through GK) domains, using the most similar transcript variants available, indicated the zebrafish β2.1, *Fugu *and *Tetraodon *β2 genes shared ~87% identity with other vertebrate β2 genes (Figs. 2C; see Fig S3 for alignment of core regions). We extended this analysis to five teleost species by including ESTs or proteins derived from genomic sequences. Three crystallographic studies on mammalian CACNB genes have identified a total of 21 amino acid residues as critical for interaction of the β subunit with the α subunit AID (alpha interaction domain). In our five different teleost species, we observed that all 21 residues were nearly 100% conserved with mammals (see Additional File 3) [48-50].

**Figure 3:**
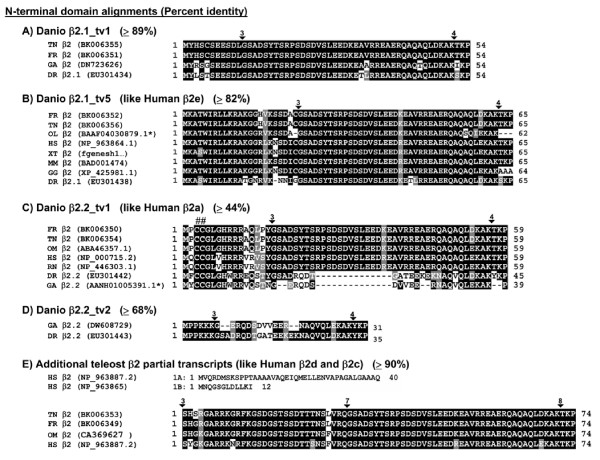
**Sequences and alignments of alternatively spliced 5' regions**. (A -E) Alignments show the entire N-terminal portion of the protein (sequences prior to the SH3 domain) for vertebrate β2 sequences culled from public databases. An overall percent amino acid identity, calculated for the various pairwise combinations, is listed in parentheses for each peptide alignment. Where appropriate, homology to previously described human transcript variants is indicated [57]. E) In humans, exon 1A or exon 1B is spliced to exon 2A. No exon 1A or1B-like exons could be identified in the current databases for teleosts, but an exon homologous to human 2A is present in several species. DR, *Danio rerio*; FR, *Fugu rubripes*; GA, *Gasterosteus aculeatus *(three-spined stickleback); GG, *Gallus gallus*; HS, *Homo sapiens*; MM, *Mus musculus*; OL, *Oryzias latipes *(Medaka killifish); OM, *Onchorhynchus mykiss *(trout); RN, *Rattus norvegicus*, TN, *Tetraodon nigroviridis*; XT, *Xenopus tropicalis*. In this and other alignments, conceptual translations were used if protein accession numbers were not available. * denotes a single genomic contig which contains the predicted exons shown. 6 denotes an exon border, numbered with reference to (A-D) zebrafish or (E) pufferfish exons. ##, indicates the location of two conserved cysteine residues which are palmitoylated in human β2 proteins.

In contrast, the more divergent zebrafish β2.2 core region shares only ~62% amino acid identity (see Additional File 4 for alignment of core regions). The most extreme sequence divergence in the zebrafish β2.2 gene occurs at the 5' end of the gene, both 5' of and within the SH3 domain, although high levels of divergence exist throughout the entire protein. We identified a single EST, termed DW608729, from three-spined stickleback as a possible ortholog to the zebrafish β2.2 gene. Within the core domain, DW608729 is 74% identical to zebrafish β2.2 (EU301442) but only 52% identical to zebrafish β2.1 (EU301434). In addition, DW608729 maps to a genomic contig, AANH01005391.1, which contains sequences homologous to zebrafish β2.2 exon 1 (encoding MFCCGLGHWRREQSTY) and β2.2 exon 2 (encoding MPPKKK)(Fig. 3). In the β2.2 gene(s), regions of high divergence encompass sequences both within and outside of inferred secondary structures [32]. Nevertheless, 19 of 21 β subunit residues noted for interaction with the α subunit AID are conserved in zebrafish β2.2 (see Additional File 4) [48-50]. High sequence divergence in β2.2 relative to other vertebrate β genes is reflected in the branch lengths on the phylogeny (Fig. 6). This pattern contrasts with that seen in the β4 group, where the two zebrafish paralogs have experienced similar rates of amino acid substitution.

Alignment of residues in the C terminus of the β subunit genes shows that sequences 3' to the GK domain are not highly conserved even among the teleost species (see Additional File 5). It has previously been noted among multiple vertebrate β subunit genes that the C terminal regions are not highly conserved. Specific functions for C terminal domains are poorly defined, although studies of truncated β2 proteins suggest the C terminus could contribute to protein function [35].

### Alternative splicing at the N terminus and within the HOOK domain

The zebrafish β2.1 and β2.2 genes undergo alternative splicing within the N-terminus and within the internal HOOK domain (Figs. 1, 2A, and 2D). The β2.1 and β2.2 genes each encode two mutually exclusive N termini (Fig. 3). Zebrafish β2.1 and β2.2, as well as the *Tetraodon *and *Fugu *β2 genes, share some 5' exons in common with mammals (Fig. 2A, lines 4 and 8; Fig. 3). In contrast, the β2.1 exon 1 sequence could not be found in any mammalian, *Xenopus *or chick databases, but was present in the *Tetraodon *and *Fugu *genomes, suggesting this exon may be specific to teleosts (Fig. 2A, line 6). The β2.2 exon 2 was found only in one other sequence, the stickleback EST DW608729, although the small size of this exon (6 amino acids) could make it difficult to recognize if it had diverged in other species (Fig. 2A, line 5). Additional 5' exons occur in mammals that were not observed in any teleost cDNAs or in genomic sequences available to date, although the limitation of this analysis is that complete genomic sequences are not available for all teleost species examined, and that our data relies in part on genome mining rather than extensive cDNA analysis. However, our data support the hypothesis that each of the teleost β2 loci have evolved to contain a unique combination of 5' exons, each of which is predicted to encode an alternative protein N-terminus.

**Figure 4:**
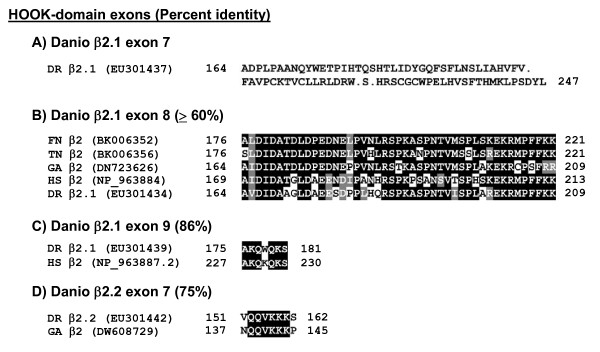
**Sequences and alignments of alternatively spliced exons contributing to the HOOK domain**. Four distinct exons occur in zebrafish β2 transcript variants which differentially join to exon 6 to encode the HOOK domain (see Fig. 1C). (A-C) Three of these exons are alternatively spliced in β2.1, whereas the sequence in (D) was the sole sequence found in all β2.2 transcripts. Species names are abbreviated as in Figure 3.

Alternative splicing also occurs internally for one zebrafish β2 subunit gene. β2.1 encodes three alternatively spliced HOOK domain exons (exons 7, 8 and 9; Figs. 1 and 4). The high conservation of β2.1 exons 8 and 9 with mammalian counterparts (Fig. 2A, lines 15 and 16), and exon 8 with several other teleost genes (Fig. 4), suggests that these internal sequences could have functional relevance. The β2.1 exon 7, which appears to be unique to zebrafish, includes a premature in-frame stop codon expected to truncate the protein in the HOOK domain (Fig. 2A, line 14). In β2.1 transcripts that contain both exon 8 and 9, the reading frame is altered such that a premature in-frame stop codon truncates the protein in exon 10.

No alternative splicing was observed for β2.2 transcripts in the HOOK domain; instead, all variants encode a short exon (exon 7) specific to fish that contains several positively charged residues (Fig. 2A, line 13). Likewise, the *Tetraodon *and *Fugu *genomes contain only one recognizably homologous exon (exon 9), similar to human exon 11 (Fig. 2B, line 15). The variety of β2 transcript variants is expected to encode an array of different β2 subunit proteins in teleosts.

### The large size of 5' introns

**Figure 5:**
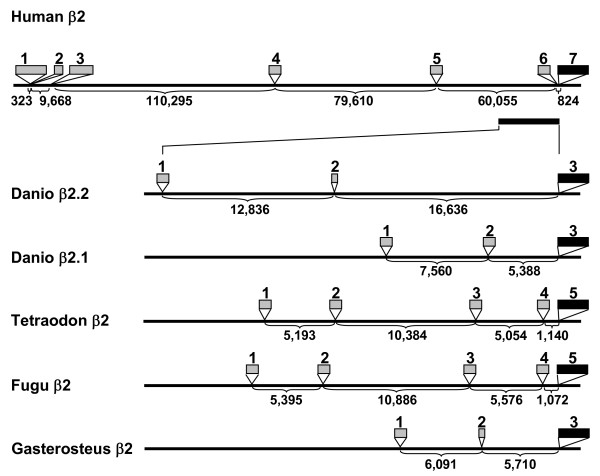
**Large intron sizes in β2 genomic loci**. Boxes denote the location of exons that encode the 5' region of β2 subunit genes. The intervening regions denote the relative sizes of introns, labeled in bp. The scale is expanded for the teleost gene diagrams relative to human. The various N-terminal (grey) exons splice to the solid black exon, which begins with amino acids GSAD...The following genomic contigs were used for the analysis: Human β2 (NT_008705.15), *Danio *β2.2 (NC_007113), *Danio *β2.1 (NC_007133), *Tetraodon *β2 (CAAE01015017.1), *Fugu *β2 (CAAB01000004.1), *Gasterosteus *β2 (AANH01005391.1, using the EST DW608729, which resembles zebrafish β2.2).

Remarkably, the introns that separate the first few 5' exons of the β2 loci are among the largest introns known in the zebrafish, pufferfish or human genomes. Each *Tetraodon *and *Fugu *β2 locus contains one intron over 10 kb, a size that ranks within the top 5% of the largest introns in pufferfish [51]. In addition, the two pufferfish genes, the stickleback β2.2-like gene, and both zebrafish β2.2 and β2.1 each contain introns of > 5000 bp that separate 5'-most exons (Fig. 5). In pufferfish, the modal value for intron size is 79 bp, with 75% of introns < 425 bp in length [51]. Given the compact nature of the pufferfish genomes [52], it was not surprising that introns of the human β2 locus exceeded the size of those in fish. Nevertheless, the trend of megasized introns in the N-terminus of β2 loci extends to the human genome. Three giant introns ranging in size from 60–100 kb each separate the human β2 exons in the N-terminus. Thus, when human exon 3 is spliced to exon 7 (beginning GSAD...), the splicing machinery must exclude over 250 kb of intronic sequence. In contrast, introns in the remaining part of the human β2 gene averaged 3261 bp in size. In humans, the mean size of introns adjoining coding sequences is 3749 bp, but 75% of introns are smaller than 2609 bp [51,53,54]. A recent analysis of the genomes of *Arabidopsis thaliana*, *Drosophila*, mouse and human indicated that the median sizes of introns separating 5' UTR (non-coding) sequence are significantly larger than introns separating coding sequence [54]. Even so, the median size of human 5' UTR introns (8223 bp, [54]) is still much smaller than the human 5' β2 introns, which separate coding sequences.

### Divergence of the calcium channel β2 genes in zebrafish

**Figure 6:**
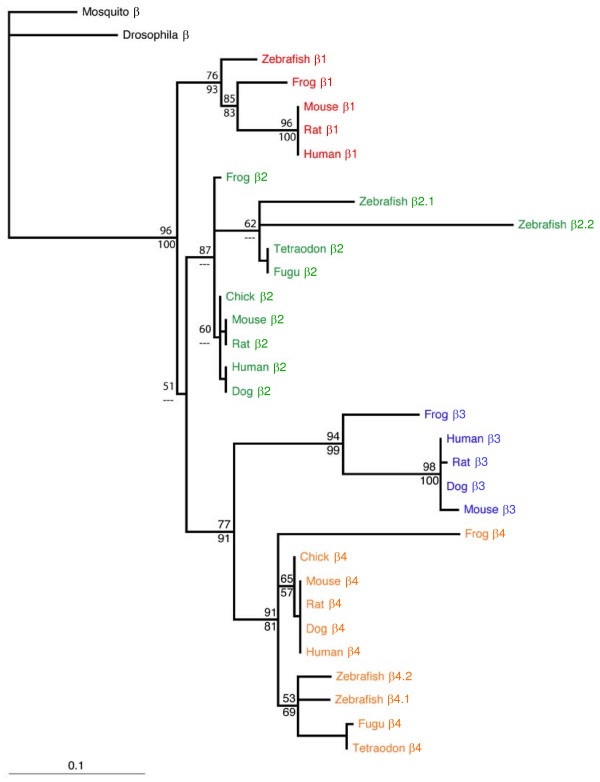
**Phylogeny of β2 subunit genes**. Phylogenetic tree showing the relationships among β2 subunit core domain (SH3 – GK) sequences. Numbers above the nodes indicate maximum likelihood quartet puzzling support values; numbers below the nodes are maximum parsimony bootstrap proportions. "---" indicates a node that was unresolved in the maximum parsimony analysis. The long branch associated with zebrafish β2.2 reflects an elevated rate of amino acid substitution throughout the core domain, particularly at the 5' end. See Methods for accession numbers of sequences used.

We used CLUSTALW software to align the β2 subunit core domain amino acid sequences and TreePuzzle and PAUP* to construct phylogenetic trees using maximum likelihood and maximum parsimony, respectively [55]. By using only core domains (~SH3-GK), we minimized differences due to alternative splicing (Fig. 6). The *Fugu *and *Tetraodon *β2 subunit genes, zebrafish β2.1, zebrafish β2.2, and other vertebrate β2 genes form a monophyletic group (MLQP = 87%, MPBP < 50%). The sequence of β2.2 is substantially more diverged from other vertebrate β2 genes than is β2.1, both in amino acid replacements (see branch lengths in Fig. 6) and insertions/deletions. Portions of the gene were sufficiently divergent to be essentially random in sequence with respect to all other taxa, and the region 5' of the SH3 domain also contained an 18-amino acid deletion. No convincing region of synteny could be established between any of the teleost β2 genes and human β2, despite the fact that human chromosome 10 and mouse chromosome 2 share a region of synteny inclusive of the β2 genes. Thus, a syntenic approach was not useful to confirm or refute the orthology of zebrafish β2.1 or β2.2 with mammalian β2 genes.

### Heterogeneity of calcium channel β2 expression in the embryo and adult

**Figure 7:**
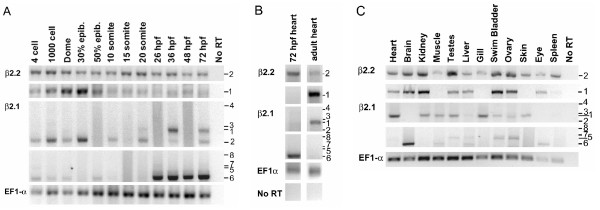
**Expression of β2 subunit transcript variants in the embryo and adult**. RT-PCR analysis using transcript variant-specific primers (located in the 5' exons 1 or 2 and exon 10) was performed on RNA samples from A) whole embryos at various developmental stages, B) cardiac tissue dissected from *cmlc2:GFP *embryos or from adult fish, and C) adult organs and tissues. Expression of a housekeeping gene, EF1α, was used as a control for RNA integrity. In B, 72 hpf or adult RNA reactions were run on single gels, subsequently subdivided to multiple panels for clarity in presentation. Transcript variant numbers are listed to the right of panels; refer to Fig. 1D.

To determine whether β2 genes are expressed in a stage- or tissue-specific manner in early embryogenesis, we performed RT-PCR on RNA isolated from embryos of several stages in early development. To track the expression patterns of specific transcript variants (Fig. 2D), we used forward primers specific to the 5' exons 1 or 2, and reverse primers in exon 10 (the GK domain) in RT-PCR experiments (Fig. 7). Surprisingly, amplification of β2.1 and β2.2 transcripts occurred in embryos as young as the 4-cell and 1000-cell stages (Fig. 7A). Since zebrafish zygotic transcription does not initiate until the 10^th ^cell division (~the 1000-cell stage) [56], the presence of mRNA in 4-cell embryos indicates that the transcripts are of maternal origin. The β2.2 transcript variant 2 was expressed steadily from the 4-cell stage through 72 hours post-fertilization (hpf). In contrast, β2.2 transcript variant 1 showed a pulse of expression in early epiboly stages. β2.1 transcript variant 6 was robustly detected from 26 hpf through at least 3 dpf. Other β2.1 transcript variants (1 and 2) were detected more sporadically or were undetectable in this assay, consistent with their rare recovery in RACE reactions. Thus, both β2.1 and β2.2 are expressed from the earliest stages of embryogenesis, but show significant heterogeneity in patterns of transcript variant expression throughout the first three days of embryogenesis.

To determine which transcript variants of the zebrafish β2 genes are expressed in the embryonic heart, we performed RT-PCR on RNA extracted from cardiac tissue at 72 hpf (Fig. 7B). These data indicate that only a subset of β2.1 and β2.2 transcript variants are expressed in the embryonic heart relative to the adult heart. In humans, at least seven β2 transcript variants are expressed in the heart, including β2a and β2e [34,35,39]. In zebrafish, the β2.1 transcript variant 6 (resembling β2e at the N-terminus) was expressed only in the embryo, whereas the β2.2 transcript variant 1 (resembling human β2a at the N-terminus) was expressed in the adult but not embryonic heart.

The expression of β2 subunits in several adult organs or tissues was assayed. In most adult tissues, we observed transcript variant-specific patterns of expression for the β2.1 and β2.2 genes (Fig. 7C). Mutually exclusive expression of β2.1 transcript variants 1 and 6 occurred in the heart and brain, respectively. Some other tissues express more than one β2.1 transcript variant. Many adult tissues express both β2.2 transcript variants 1 and 2, but a few tissues (muscle, gill and skin) expressed only β2.2 transcript variant 2. Thus, adult tissues also show significant heterogeneity in expression of β2 subunit transcript variants.

## Discussion

### Teleost calcium channel β subunits and embryonic development

We have identified four new calcium channel β2 subunits in teleosts, including two in zebrafish and one gene each in the *Fugu *and *Tetraodon *genomes. Like the mammalian calcium channel β subunits, the four teleost β2 subunit genes encode characteristic MAGUK family proteins, with SH3 and GK domains that are highly conserved with other vertebrates. We show that several alternatively spliced transcripts arise from both β2.1 and β2.2 subunits, which are expressed in the embryo and adult but subject to both temporal and spatial regulation. Only selected transcript variants of each β2 subunit gene are expressed in the embryonic heart, and these differ from adult transcript variants. Thus, the heterogeneity of β subunits and their transcript variants in teleost species is extensive.

### Gene structure of teleost calcium channel β2 subunits

Alternative splicing occurs within the N-terminus as well as internally in the β2.1 and β2.2 subunit genes. Notably, each zebrafish β2 subunit gene encodes at least two mutually exclusive 5' exons, each with a separate translation initiation site. Each gene has one 5' exon conserved with mammals, and one 5' exon unique to fish. Similarly, some of the internal alternatively spliced exons are similar to those in mammals, while others are unique to teleosts. This variety of transcript variants potentially encodes an array of different β2 subunit proteins. Previous studies have demonstrated that 5' variation due to alternative splicing can be functionally significant in β subunits. When expressed in HEK 293 cells, human 5' β2 subunit variants β2a and β2e (starting with human exons 4 or 6, respectively) showed differential sub-cellular localization compared to transcript variants β2b, β2c and β2d (starting with human exons 4, 1 and 2, respectively) [57]. Moreover, the five human 5' β2 subunit variants differentially affected open probability, peak current and availability of L-type channels [57,58]. Likewise, in *Xenopus *oocyte expression studies, human β4 subunits with alternatively spliced N-termini showed functionally distinct electrophysiological properties [59,60]. Domain swapping experiments further indicate the functional significance of the N-terminal sequences in β subunit proteins. Replacing the β1b N-terminus with β2a N-terminus created a chimeric β protein with slow inactivation kinetics [61]. Conversely, replacing β2a N-terminus with the non-palmitoylated β3 N-terminus created a chimeric β protein with β3-like inactivation kinetics [62]. Post-translational modifications such as phosphorylation or palmitoylation, present on exons near the N-terminus, may account for some of the functional diversity, although this functional relationship has been experimentally demonstrated for only a few genes to date [63,64]. Intriguingly, a recent report indicates that the length of the β2 N-terminus is, independent of sequence, an important factor in mediating the magnitude of channel modulation [58]. Further functional studies using the zebrafish model system will be necessary to determine whether the array of zebrafish β2 subunit proteins indeed have different biochemical functions within the cell.

A curious feature of at least five species of teleost β2 subunit genes, as well as the human β2 subunit gene, is that some of the largest introns of the genome separate the several short, alternatively spliced 5' coding exons. Do these large introns have any adaptive significance for β2 subunit biology? In several instances, introns have been shown to incorporate enhancer or repressor elements that influence transcription [65]. Expanding this idea, we propose that the unusual β2 gene structure may provide a mechanism for independent cis-regulation of each transcript variant; that is, sequences within individual introns might independently direct the tissue and temporally-specific expression of transcript variants utilizing the associated ATG-containing 5' exon. This hypothesis is supported by the observation that transcript variant-specific patterns of expression do in fact occur in both embryo and adult. However, it is increasingly becoming appreciated that, beyond regulatory elements, introns can impact mRNA metabolism in a number of other ways. Potential effects include modulating transcription rates by regulating DNA accessibility, modulating editing and polyadenylation of the pre-mRNA, and affecting nuclear export, translation and mRNA decay rates [65,66].

The inclusion of different subsets of 5' exons (and their associated introns) among each of the pufferfish or zebrafish β2 subunit genes is interesting from an evolutionary standpoint. A canonical view is that variations in protein structure and function form the basis of evolutionary innovation and phenotypic divergence. However, as more genomes are sequenced and annotated, it is becoming evident that alternative splicing substantially increases the proteome in many species. (For example, about 40–50% of human and mouse genes contain alternative promoters [67]). A corollary to this hypothesis is that the regulatory circuitry regulating alternative splicing or the expression of particular transcript variants is also an important source of evolutionary innovation [67,68]. The mechanisms by which new introns arise during evolution, and the impact of large intron size per se on cis-regulation or other adaptive phenomena, remain actively debated areas of research [69-71].

### The evolutionary origin of the duplicated calcium channel β2 subunit genes

Zebrafish β2 duplication may be the consequence of a genome-wide duplication thought to have occurred in teleosts approximately 300 million years ago [72-74]. Alternatively, duplication could be the result of an ancient region-specific duplication. The β2 subunit gene does not appear to be duplicated in either the *Fugu *or *Tetraodon *genomes. The existence of single β2 subunit homologs was of itself not particularly surprising since phylogeny and synteny data suggest that the common ancestor of zebrafish and pufferfish underwent a large-scale gene or complete genome duplication event [75]. Subsequently, pufferfish may have lost many duplicates that were retained in the zebrafish [75]. Data from the other β gene paralog groups (β1 and β4) are equivocal, as zebrafish have one described copy of β1 and two copies of β4 [41,76]. The fate of most duplicated genes is the accumulation of degenerative mutations in coding or regulatory regions that leads to gene loss or silencing [73]. Alternatively, genes may acquire separable patterns of expression or separable functions that then require the maintenance of functional copies of both genes in the genome (subfunctionalization) [73]. Since the two zebrafish β2 genes are both robustly but differentially expressed, our data are more consistent with subfunctionalization.

### Developmental expression patterns of calcium channel β2 subunit transcript variants

Calcium is an important signal in the embryo even prior to gastrulation [77]. Calcium gradients, waves and pulses have been described in the blastula and early gastrula that may represent key pattern forming events. The mechanism underlying these calcium signals, and whether they encompass voltage-gated calcium channels, is not clear. Surprisingly, both β2 subunits show maternal as well as the early zygotic expression of several transcript variants. Our recent study of two zebrafish β4 subunits, which are also expressed maternally and zygotically in the gastrulating embryo, indicates that these genes are essential for normal epiboly. Initially, we predicted that β subunits might be involved in the gastrulating embryo voltage-gated calcium channel-related functions. However, our study showed that the zebrafish β4 subunits, at least, operate independently of voltage-gated calcium channels in mediating epiboly [76].

In addition to possible early roles, we find that expression of β2.1 or β2.2 is under strong temporal control in the post-gastrula embryo and in adult tissues. Of particular interest, given the cardiac lethality observed in mouse β2-deficient embryos, is the observation of differential subsets of β2.1 and β2.2 transcript variants expressed in the embryonic and adult heart. This observation supports the hypothesis, previously proposed by others, that the heterogeneity of β subunit transcript variant expression within the heart may provide a mechanism for fine-tuning the cardiac voltage-activated current as the organism progresses through embryonic, juvenile and adult stages [38,39]. In addition, the expression levels of particular β2 transcript variants has also been linked to pathophysiology of heart failure in humans [78-80]. Clearly, the first step in interpreting the function of β subunits in any model system is simply to understand when and where the various transcript variants are expressed.

## Conclusion

The primary novel findings of this study are as follows: 1) We have cloned two new β2 subunit genes in zebrafish, one of which is phylogenetically quite divergent in amino acid sequence. They are classified as MAGUK proteins on the basis of high conservation with mammalian MAGUK core regions. 2) Alternative splicing occurs at the β2 N-termini and internally. A comparative analysis showed that a subset of 5' exons present in several teleosts species is shared with mammals, while a different subset appears to be unique to teleosts. 3) Some of the largest introns in the human or teleost genomes separate the small, alternatively spliced 5' exons of β2 genes, leading us to hypothesize that β subunit transcript variant expression may be under independent cis-regulatory control. 4) The zebrafish β2 genes are expressed maternally and zygotically in the gastrulating embryo and show strong evidence of temporal and spatial regulation in embryogenesis and in adults. 5) Only a subset of β2 subunit transcript variants is expressed in the embryonic heart, and they differ from those expressed in the adult heart. In sum, this work provides the groundwork for a study of functional aspects of β2 subunit biology. The high degree of temporal and spatial control of β subunits, combined with recently identified non-canonical functions for particular β subunits or transcript variants, suggests that a functional analysis of these genes would provide intriguing insight.

## Methods

### Zebrafish strains and care

This study used the WIK zebrafish strain. Zebrafish care was provided in accordance with animal care policies of Colorado State University. Embryos were staged as described [81].

### Isolation of zebrafish β2 cDNAs

Total RNA was prepared from embryos aged 24–72 hpf using Trizol (Invitrogen, Carlsbad, CA) as per manufactuer's protocol. Poly(A)^+ ^mRNA was prepared using magnetic beads (μMACS mRNA kit Miltenyi Biotec), and resuspended in RNase free dH_2_O at a concentration of 1 mg/ml. RNA was stored in aliquots as an ethanol precipitate at -80 degrees Celsius. RACE Ready cDNA was created using the SMART RACE cDNA synthesis kit (Clontech, Palo Alto, CA). RACE reactions were carried out as per manufacturer's protocol using gene-specific primers designed to hybridize within the SH3 and GK domain encoding regions of β subunit genes identified by BLAST searches of the zebrafish genome (Fig. 1 and Additional File 2A). RACE PCR products were separated in ethidium bromide-stained agarose gels, excised and purified over a silica matrix column (Zymoclean Gel DNA Recovery Kit, Zymo Research). cDNA fragments were cloned into pCR 2.1-TOPO using the TOPO TA cloning method (Invitrogen, Carlsbad, CA).

### Pufferfish sequence identification and amino acid alignments

We used human and zebrafish β2 sequences to identify homologous *Fugu *and *Tetraodon *β2 sequences by a multi-step process. First, we performed protein vs. translated DNA (TBLASTN) searches using human or zebrafish β2 sequences against the NCBI, Sanger Centre, UCSC Genome Bioinformatics and pufferfish genome databases [43-47]. This process identified the best matches among the *Fugu *or *Tetraodon *predicted proteins derived from contig assemblies and other sequences available for pufferfish. The homologous predicted *Fugu *or *Tetraodon *proteins were re-tested by TBLASTN against mammalian Refseq proteins to confirm they were more similar to β2 subunit genes than other β subunit family members. Next, we used the homologous *Fugu *or *Tetraodon *seqeunces in BLAT searches of UCSC Genome Bioinformatics database sequences to identify the genomic location of *Fugu *or *Tetraodon *β2 exons 3–14 and to determine conserved splice donor and splice acceptor sites [45]. To identify the short N terminal exons (exons 1 and 2) or search for alternative HOOK domain exons in pufferfish, we used zebrafish or human exons in 1) BLAT searches of UCSC Genome Bioinformatics pufferfish (*Fugu *or *Tetraodon*) sequences, 2) MEGABLAST, peptide motif and BLASTP searches of NCBI, and 3) BLAST or BLAT searches of the Fugu Genome Browser, the Tetraodon Genome Browser, or the Sanger Center Ensemble Genomic databases [43-47]. Exons were numbered according to their sequential location in the genome, and probable splice donor and splice acceptor sites were identified for each exon. The pufferfish sequences were submitted to the Third Party Annotation database of NCBI. Additional teleost sequences from trout, medaka, and stickleback were obtained by searching the NCBI EST or WGS databases, or from JGI [82].

### Phylogenetic analysis

Amino acid sequences representing the β subunit "core" (i.e., from the amino acids "GSAD" just prior to the SH3 domain through the end of the GK domain) were aligned using ClustalW, varying gap opening and extension parameters; regions for which reliable homology could not be established because of indels were excluded from analysis [83]. Portions of the zebrafish β2.2 gene were sufficiently divergent in sequence from all other genes (including those from *Drosophila *and mosquito) to be essentially random. To minimize the phylogenetic analytical problems associated with such extreme rate variation among lineages, we excluded regions of the alignment in which zebrafish β2.2 contained autapomorphic amino acid substitutions for ≥ 3 adjacent amino acid positions. Such exclusions resulted in an alignment of 252 amino acids. Maximum likelihood analyses were implemented in TreePuzzle [84] using the Müller & Vingron (VT) model of amino acid substitution with a mixed model of rate heterogeneity; amino acid frequencies, the gamma distribution parameter alpha, and the proportion of invariant sites were estimated from the data [85]. Nodal support was assessed using quartet puzzling. Equally-weighted maximum parsimony analyses were carried out using PAUP* [55]. A heuristic search was performed with 25 random additional replicates and TBR branch-swapping. Bootstrap proportions for clades were assessed with 1,000 pseudo-replicates. Resulting trees were rooted with *Drosophila *and mosquito. Teleost sequences other than pufferfish and zebrafish were not included because they only span a portion of the core domain.

### Reverse transcription assays

As an RNA source, we used whole embryos, or individual organs or tissues dissected from adults aged approximately 1 year. Cardiac tissue from embryos was isolated on the basis of its GFP expression from the *cmlc2:GFP *transgenic line, in which the *cmlc2 *promoter drives expression of GFP only in the heart [86]. RNA was extracted using the Trizol method and stored at -80°C. RT-PCR was performed for β2.1 and β2.2 genes using the Access RT-PCR System (Promega) using high-fidelity polymerases. The thermal cycling program was as follows: 48°C for 45 minutes (reverse transcription), followed by 94°C for 2 minutes (activation of PCR enzyme) and 40 cycles of 94°C for 30 seconds, 60°C for 1 minute, and 68°C for 2 minutes. A final step of 68°C for 7 minutes allowed for a final extension. Electrophoresis of the final product was performed on a 2% agarose gel containing ethidium bromide. Gels were imaged using a digital camera and imaging software (Scion Corporation, Frederick, MD).

### Accession numbers

The following Genbank sequences were used for the phylogenetic analysis: CACNB1 genes: NP_000714.3, NP_660099.1, NP_059042.1, AAH76523.1; CACNB2 genes: EU301442 (mRNA),EU301434 (mRNA), NP_963864.1, NP_075605.1, XP_855731.1, NP_446303.1, XM_425981.1, BK006353, BK006349; CACNB3 genes: NP_000716.2, XP_543689.2, NP_031607.1, NP_036960.1, AAA75519.1; CACNB4 genes: ABU93250.1, ABU93253.1, XP_851697.1, NP_000717.2, NP_001032176.1, NP_001001733.1, A45982, CAG08264.1; other CACNB genes: NP_995685.1, XP_317433.2, NP_491193.1. In addition, if no appropriate sequence was available in Genbank, we used sequences from JGI database [82], including *X. tropicalis *genome assembly version 4.1, entitled FGENESH1_PM.C_SCAFFOLD_1068000001 (*Xenopus *CACNβ2); C_scaffold_227000005 (*Xenopus *CACNβ4); and e_gw1.686.1.1(*Xenopus *CACNβ1). From the *Fugu rubripes *genome assembly version 4.0, we used fgh5_pm.C_scaffold_38000036 (*Fugu *CACNβ4). The JGI sequences are available upon request from the authors.

## Abbreviations

Reverse-transcriptase polymerase chain reaction (RT-PCR), membrane-associated guanylate-kinase protein (MAGUK), voltage-dependent L-type Ca^2+ ^channel (V-LTCC), rapid amplification of cDNA ends (RACE), basepairs (bp), cardiac myosin light chain (cmlc), green fluorescent protein (GFP), days post-fertilization (dpf), hours post-fertilization (hpf), maximum parsimony bootstrap proportion (MPBP), maximum likelihood quartet puzzling (MLQP).

## Authors' contributions

AS generated the cDNA used for RACE experiments; AME, CAM and DMG carried out the RACE and RT-PCR cloning experiments; AME carried out the RT-PCR expression experiments; DBG and DMG did the *Fugu*, *Tetraodon *and other teleost bioinformatics and alignments; RLM contributed the phylogenetic analysis; DMG wrote the manuscript with comments from all authors. All authors read and approved the final manuscript.

## Supplementary Material

Additional file 1 **Amino acid sequences of 5' exons**. The alternatively spliced 5' exons listed here occur as the first or second exon in the transcript variant. They occur in the N-terminus of the protein, prior to the beginning of the SH3 domain. For convenient comparison, human exons are co-listed, using data from [57]. See Fig. 1D for description of the transcript variants. Click here for file

Additional file 2 **RT-PCR analysis and list of primers**. (A) A list of primers used for RT-PCR analysis, and their locations (see also Figure 1 for primer locations). (B) A representative RT-PCR analysis of *Danio rerio *β2.1 and β2.2 transcripts. In lanes 1–3, a single band of the expected sizes amplified for β2.2, consistent with a lack of alternative splicing in the HOOK domain (spanning exons 5–8 in β2.2). In lanes 6 and 7, products consistent with β2.1_tv1 (697 bp) or β2.1_tv6 (580 bp) tended to amplify robustly. Potentially, larger amplicons from less abundant transcript variants may be out-competed in these reactions. We therefore tested primers closely flanking the HOOK domain (spanning exons 5–10 in β2.1). In lane 8, products consistent with all HOOK domain transcript variants were observed (a 647 bp band, a 415/394 bp doublet band, and a 280 bp band). Cloning and sequencing of these products of Lane 8 confirmed that the bands represented are the transcripts indicated. Lanes 4 and 9 are positive controls using primers to the house-keeping gene EF1α (220 bp). Lanes 5 and 10 are negative controls. Reactions were run on two gels, as shown (some lanes have been removed). (C) For each primer pair, the expected sizes of PCR amplicons and the exons that comprise them are listed. Click here for file

Additional file 3 **Amino acid alignments of zebrafish β2.1 subunit "core" regions with other vertebrate genes**. The light grey bars indicate the SH3 domain, black bars the HOOK domain, and dark grey bars the GK domain. ▼ denotes an exon border (exons numbered according to *Danio rerio *β2.1_tv1, which lacks the alternatively spliced exons 8 and 9). The plus signs (+) indicate the alpha binding pocket residues suggested by crystallographic studies to contribute side-chain contacts with the AID domain [48-50]. Note that the zebrafish isoleucine at residue 325 differs from the conserved mammalian valine, but isoleucine occurs in this position in human CACNB3 and other CACNB genes [49]. Likewise the zebrafish threonine at residue 329 is either serine or threonine in the mammalian CACNB genes [50]. The crystal structure predicts the sequences PYDVVP (just 5' to the GK domain), conserved in all genes shown, forms a beta strand that folds back to interact with the SH3 domain [50]. See Fig. 1D for description of exons present in each transcript variant. Click here for file

Additional file 4 **Amino acid alignments of the more divergent zebrafish β2.2 subunit "core" regions with a potential homolog in three-spined stickleback**. The light grey bars indicate the SH3 domain, black bars the HOOK domain, and dark grey bars the GK domain. ▼ denotes an exon border (exons numbered according to *Danio rerio *β2.2_tv1). The plus signs (+) indicate the alpha binding pocket residues that contribute side-chain contacts with the AID domain of the α subunit, whereas the minus sign (-) marks a valine residue that was not conserved in either species. Grey plus signs represent mammalian residues conserved in zebrafish but not stickleback [48-50]. Note that DW608729 EST is only a partial mRNA sequence which does not span the complete core region. See Fig. 1D for description of exons present in each transcript variant. Click here for file

Additional file 5 **Amino acid alignments of the C-terminal regions of several teleost β2 clones with human β2**. The C terminal residues aligned here reside 3' to the GK domain, and are typically encoded by the final two exons of the β2 gene. For stickleback, translations of available EST sequences mapping to the C terminal portion of the β2 gene were included, although they may not represent the full sequences encoded by the final two exons. Click here for file
