# Supplementary figures and images for: The calcium channel β2 (CACNB2) subunit repertoire in teleosts
Source: BMC Mol Biol. 2008 Apr 17;9:38. doi: 10.1186/1471-2199-9-38 (PMC2365960; doi:10.1186/1471-2199-9-38)

## Slide 1
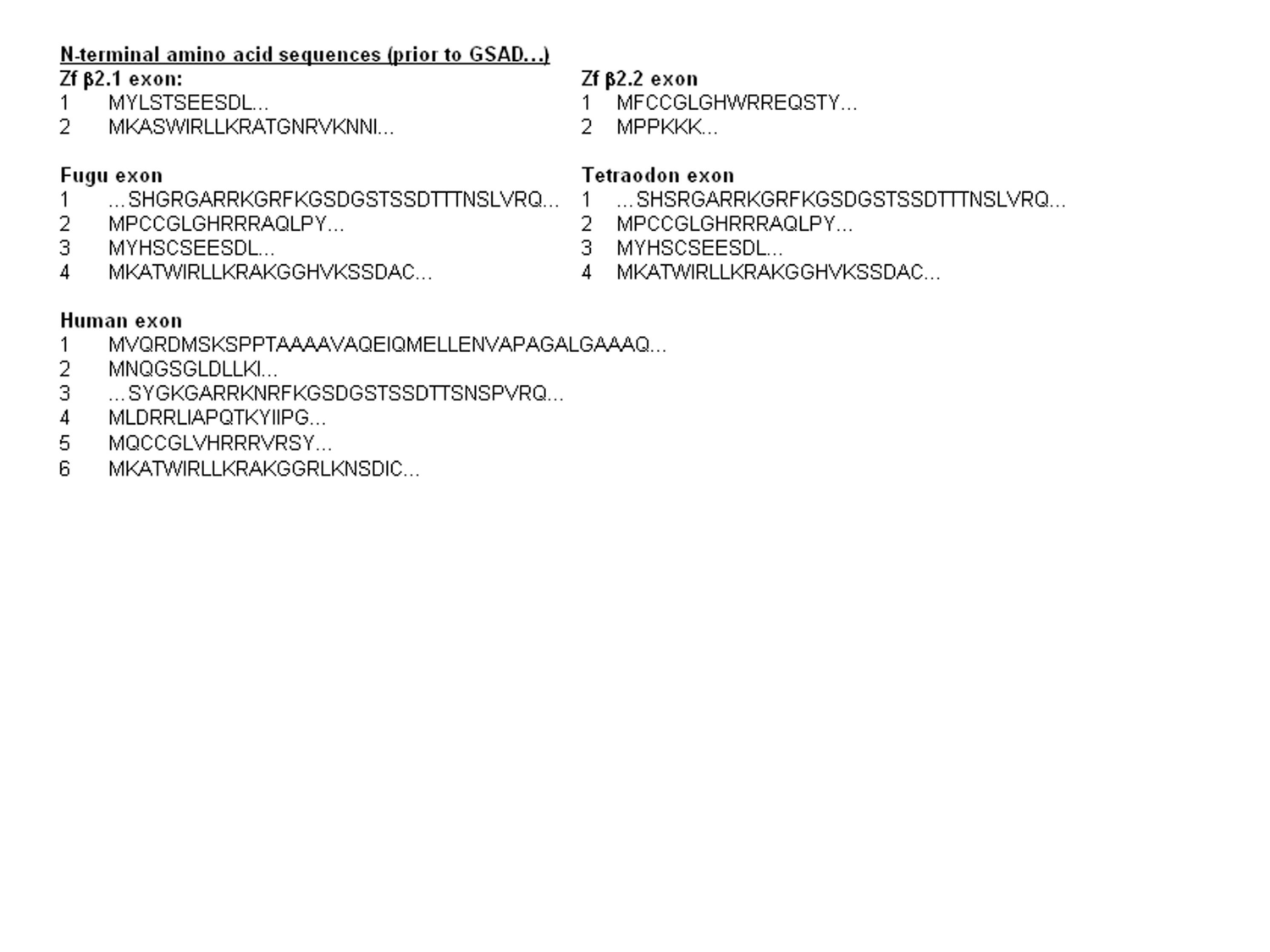

Supplement: Additional file 1 — Amino acid sequences of 5' exons. The alternatively spliced 5' exons listed here occur as the first or second exon in the transcript variant. They occur in the N-terminus of the protein, prior to the beginning of the SH3 domain. For convenient comparison, human exons are co-listed, using data from [57]. See Fig. 1D for description of the transcript variants. [file 1471-2199-9-38-S1.ppt]

## Slide 1
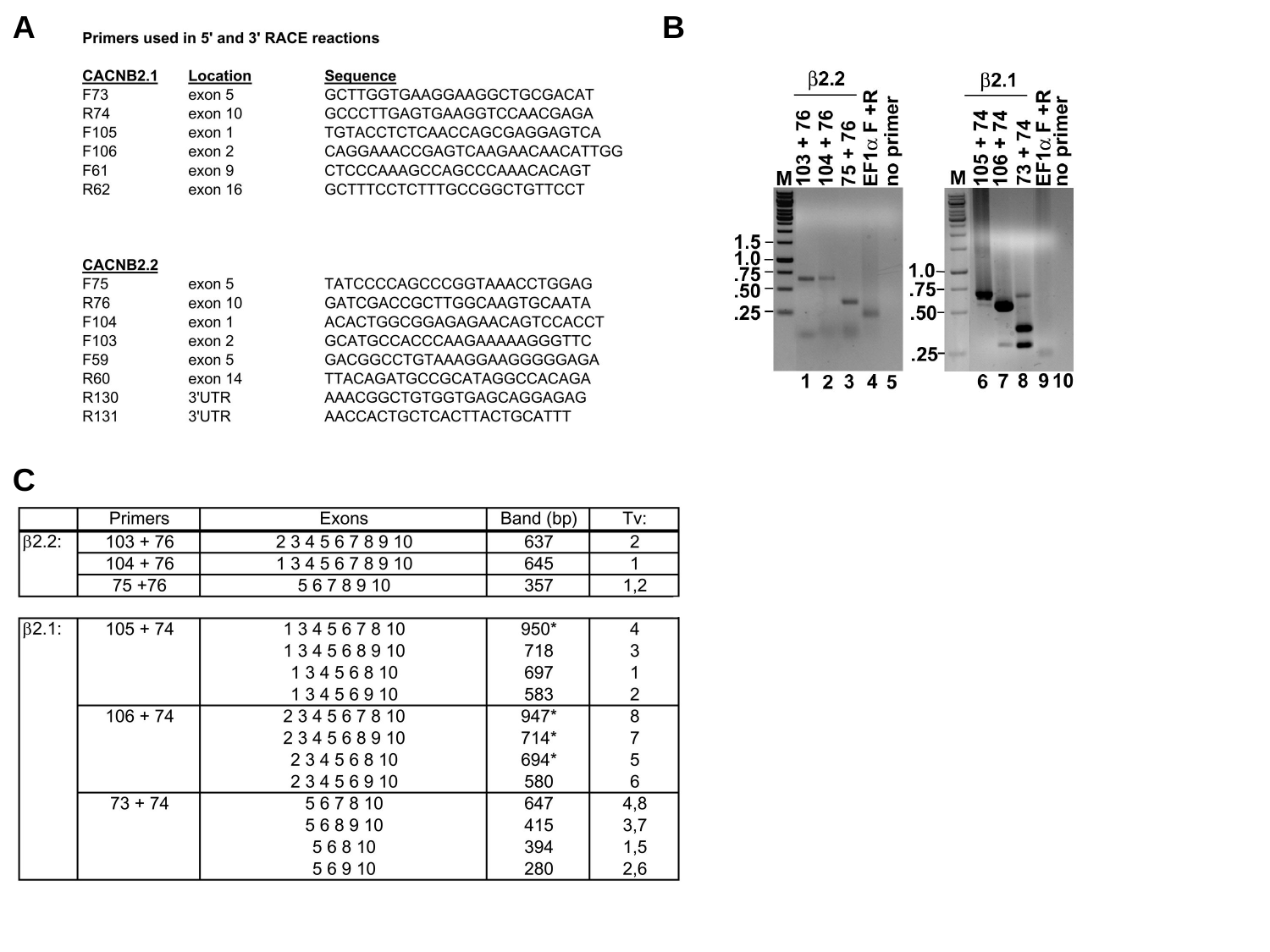

A
B
C

Supplement: Additional file 2 — RT-PCR analysis and list of primers. (A) A list of primers used for RT-PCR analysis, and their locations (see also Figure 1 for primer locations). (B) A representative RT-PCR analysis of Danio rerio β2.1 and β2.2 transcripts. In lanes 1–3, a single band of the expected sizes amplified for β2.2, consistent with a lack of alternative splicing in the HOOK domain (spanning exons 5–8 in β2.2). In lanes 6 and 7, products consistent with β2.1_tv1 (697 bp) or β2.1_tv6 (580 bp) tended to amplify robustly. Potentially, larger amplicons from less abundant transcript variants may be out-competed in these reactions. We therefore tested primers closely flanking the HOOK domain (spanning exons 5–10 in β2.1). In lane 8, products consistent with all HOOK domain transcript variants were observed (a 647 bp band, a 415/394 bp doublet band, and a 280 bp band). Cloning and sequencing of these products of Lane 8 confirmed that the bands represented are the transcripts indicated. Lanes 4 and 9 are positive controls using primers to the house-keeping gene EF1α (220 bp). Lanes 5 and 10 are negative controls. Reactions were run on two gels, as shown (some lanes have been removed). (C) For each primer pair, the expected sizes of PCR amplicons and the exons that comprise them are listed. [file 1471-2199-9-38-S2.ppt]

## Slide 1
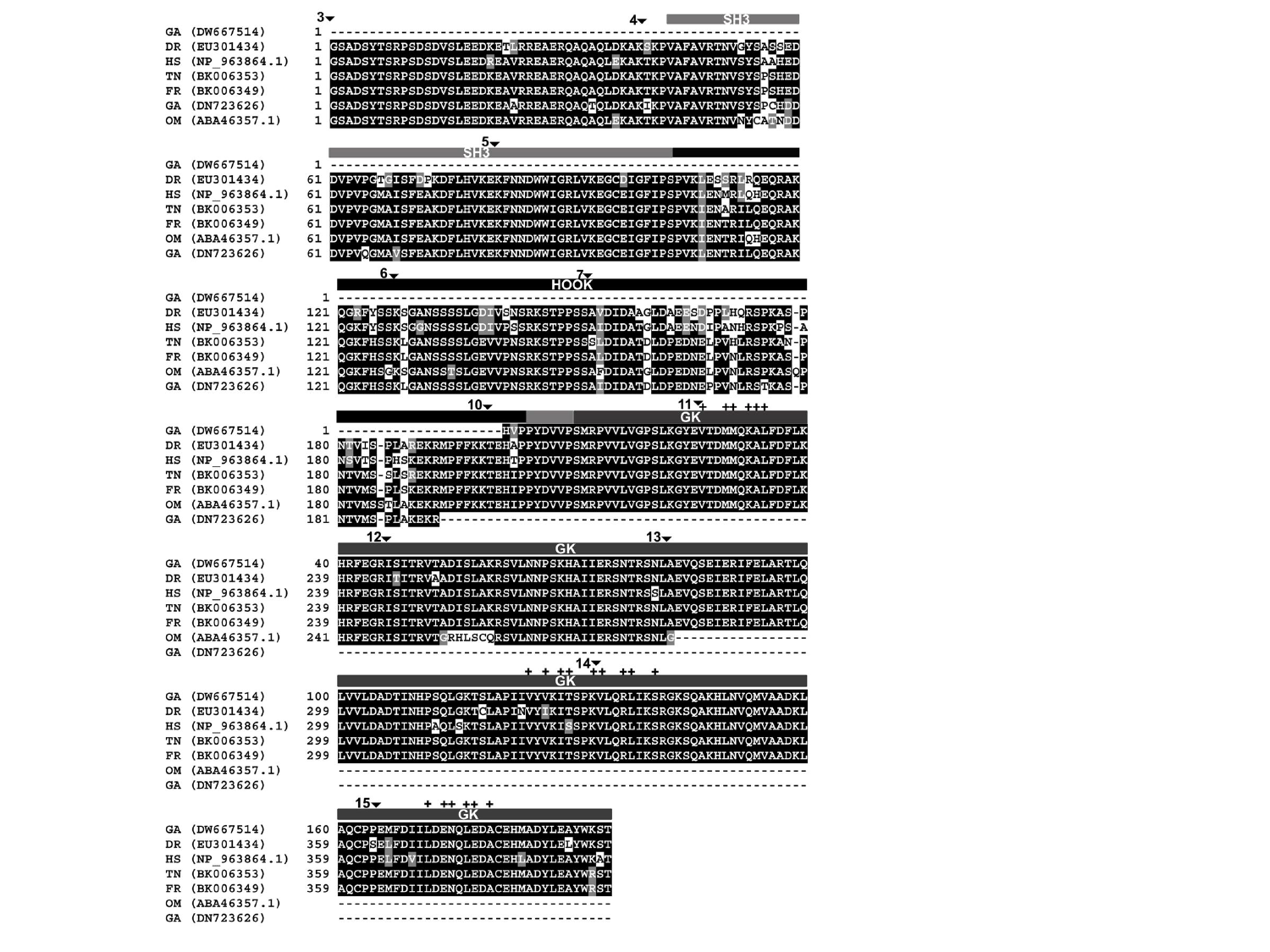

Supplement: Additional file 3 — Amino acid alignments of zebrafish β2.1 subunit "core" regions with other vertebrate genes. The light grey bars indicate the SH3 domain, black bars the HOOK domain, and dark grey bars the GK domain. ▼ denotes an exon border (exons numbered according to Danio rerio β2.1_tv1, which lacks the alternatively spliced exons 8 and 9). The plus signs (+) indicate the alpha binding pocket residues suggested by crystallographic studies to contribute side-chain contacts with the AID domain [48-50]. Note that the zebrafish isoleucine at residue 325 differs from the conserved mammalian valine, but isoleucine occurs in this position in human CACNB3 and other CACNB genes [49]. Likewise the zebrafish threonine at residue 329 is either serine or threonine in the mammalian CACNB genes [50]. The crystal structure predicts the sequences PYDVVP (just 5' to the GK domain), conserved in all genes shown, forms a beta strand that folds back to interact with the SH3 domain [50]. See Fig. 1D for description of exons present in each transcript variant. [file 1471-2199-9-38-S3.ppt]

## Slide 1
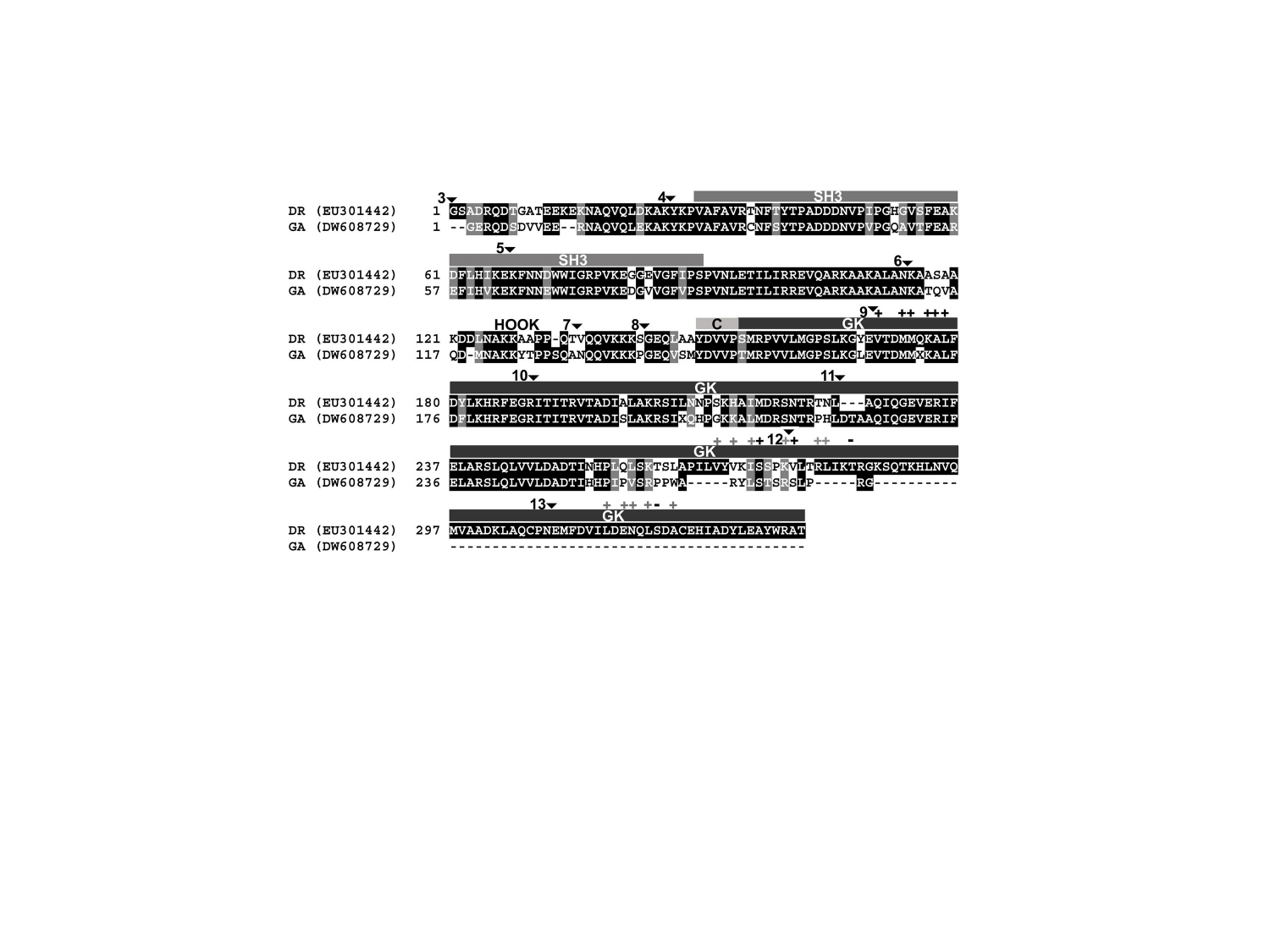

Supplement: Additional file 4 — Amino acid alignments of the more divergent zebrafish β2.2 subunit "core" regions with a potential homolog in three-spined stickleback. The light grey bars indicate the SH3 domain, black bars the HOOK domain, and dark grey bars the GK domain. ▼ denotes an exon border (exons numbered according to Danio rerio β2.2_tv1). The plus signs (+) indicate the alpha binding pocket residues that contribute side-chain contacts with the AID domain of the α subunit, whereas the minus sign (-) marks a valine residue that was not conserved in either species. Grey plus signs represent mammalian residues conserved in zebrafish but not stickleback [48-50]. Note that DW608729 EST is only a partial mRNA sequence which does not span the complete core region. See Fig. 1D for description of exons present in each transcript variant. [file 1471-2199-9-38-S4.ppt]

## Slide 1
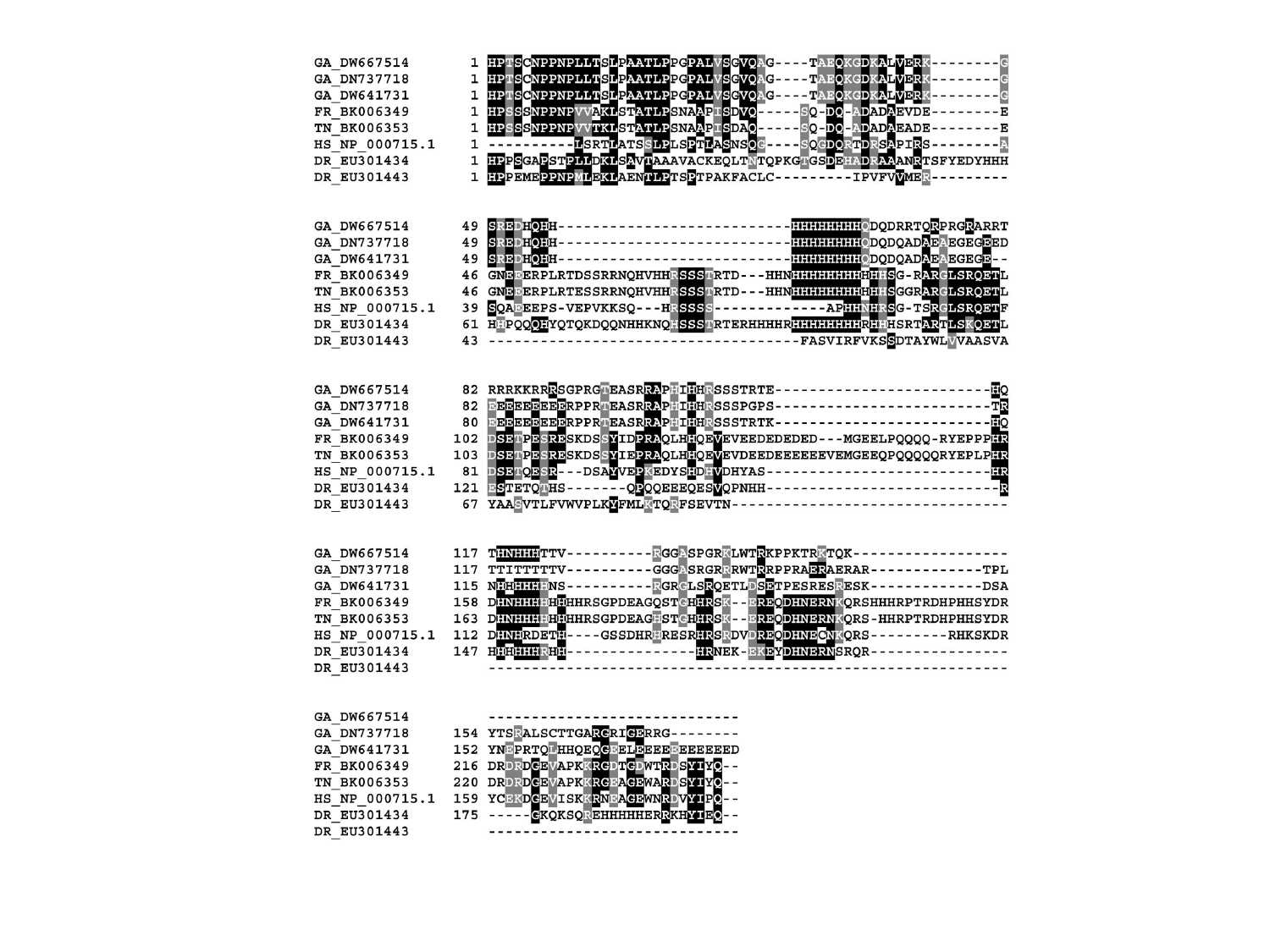

Supplement: Additional file 5 — Amino acid alignments of the C-terminal regions of several teleost β2 clones with human β2. The C terminal residues aligned here reside 3' to the GK domain, and are typically encoded by the final two exons of the β2 gene. For stickleback, translations of available EST sequences mapping to the C terminal portion of the β2 gene were included, although they may not represent the full sequences encoded by the final two exons. [file 1471-2199-9-38-S5.ppt]
